# The Potential Implications of Autonomous Vehicles in and around the Workplace

**DOI:** 10.3390/ijerph15091876

**Published:** 2018-08-30

**Authors:** Simone Pettigrew, Lin Fritschi, Richard Norman

**Affiliations:** 1 School of Psychology, Curtin University, Perth 6102, Australia; 2 School of Public Health, Curtin University, Perth 6102, Australia; lin.fritschi@curtin.edu.au (L.F.); richard.norman@curtin.edu.au (R.N.)

**Keywords:** automation, driverless cars, job creation, job losses, technology

## Abstract

The advent of autonomous vehicles is forecast to bring enormous changes to the workplace as positions primarily involving driving become progressively redundant. Little is known about public awareness of these impending changes and the potential impacts on society and individuals. This study involved a national survey of Australians and interviews with key stakeholders across multiple countries to identify major potential issues associated with vehicle automation, including in and around the workplace. Most survey respondents had concerns relating to job losses in driving occupations, while almost half anticipated increased employment in technology-related areas. Three primary themes were evident in the data from the stakeholder interviews: (1) the inevitability of the universal use of AVs and hence the immediate need for labour market planning, (2) associated potential effects on occupations that are not primarily structured around driving, and (3) the possibility of increased worker safety and enhanced commuting opportunities.

## 1. Introduction

Technological advances have long brought substantial changes to the workplace. The pace of change has escalated over time, with increasing application of robotic technologies to diverse occupations ranging from street cleaning to surgery [1]. The advent of autonomous vehicles (AVs) constitutes another major disruptive technological change that is likely to have enormous implications for many workers around the world. It is projected that globally there will be wide-scale redundancies among drivers in the trucking, taxi, ride-share, courier, and food-delivery industries, and workers in other related industries, such as warehousing and manufacturing, are also likely to be greatly affected [2,3]. In the US, the Department of Commerce estimates that one in nine workers are currently in occupations that will be affected by the introduction of AVs [4].

There are three primary ways in which AVs are being introduced around the world: (1) the implementation of autonomous forms of public transport (e.g., trains and buses), (2) ride-share companies are developing autonomous fleets, and (3) individuals are purchasing personal vehicles with autonomous features (e.g., lane keeping systems, adaptive cruise control, parking assistance, automatic braking while skidding, and blind spot and collision warning systems: [5,6,7,8]). These implementation scenarios are emerging in combination, and it has yet to be seen whether one mode will dominate or whether there will be different mixes of transport systems in different cities and countries. However, a likely ultimate scenario is that in the long term ‘mobility as a service’ (MaaS) will evolve to provide seamless door-to-door conveyance involving multiple forms of transport that are bundled together in the form of personalised mobility packages [9].

Various advantages and disadvantages are anticipated to arise from the advent of AVs. Major potential benefits include a substantial reduction in death and injury resulting from traffic accidents [10,11,12], enhanced safety for pedestrians and cyclists [13], large reductions in emissions [12,14,15], improved mobility for the elderly and disabled [16], and the liberating of parking spaces for other land uses [11]. The potential disadvantages include increased traffic congestion (resulting from increased travel overall due to enhanced availability and lower costs, along with empty cars travelling the roads when collecting their owners or returning from trips) [12,17], concerns relating to privacy, security, insurance, and liability [18], as well as job losses [4,18,19]. The extent to which these positive and negative outcomes eventuate will be highly dependent on the AV applications that are most widely implemented, with shared AVs anticipated to provide greater benefits and to be able to off-set some of the negative consequences of widespread private AV ownership [20].

Estimates of timing for the widespread use of autonomous vehicles (AVs) vary, although it is generally predicted that the next decade will see rapid advances in the development and availability of AVs and that they will be widely used by around 2040 [21]. The speed with which autonomous vehicles are evolving has resulted in society being largely unprepared for the systemic changes that will result. Public opinion surveys indicate a general perception that AVs are likely to have positive impacts on crash rates, road congestion, and available leisure time [20,22], but information is lacking on perceptions of the likely outcomes for working life. The limited evidence to date suggests that loss of jobs is a concern, but that it ranks behind other perceived problems relating to safety, liability, security, and privacy [23]. Assessing the degree to which the potential effects of AVs on employment are understood can assist in informing policies and programs designed to increase the salience of the impending changes, thereby triggering appropriate planning processes.

The aim of the present study was to investigate perceptions of the ways AVs will change work and the implications for the development of strategies by government and industry to enhance productivity while protecting workers’ rights and well-being. The following sections outline the multi-method approach that was adopted to access the views of a wide range of stakeholders including both consumers and experts. The findings from the qualitative and quantitative phases are presented, and then subsequently integrated in the Discussion section.

## 2. Materials and Methods 

This study was part of a larger project investigating the societal implications of AVs. There were two phases to the study: Study A was a national public opinion survey administered to Australians aged 16+ years (*n* = 1624) and Study B involved interviews conducted with key stakeholders (*n* = 43) in multiple countries. Results relating to other potential implications of the advent of AVs have been published previously (e.g., perceived health-related outcomes such as fewer crashes and enhanced mobility for the elderly [22,24]).

Study A used a questionnaire that was disseminated online via a large web panel provider (PureProfile). Quotas were specified to achieve a sample with an even gender split and equal representation of respondents in each of the following age categories: 16–30 years, 31–50 years, and 51+ years. The survey contained items relating to demographic characteristics, current transport behaviours, and perceptions of the potential outcomes associated with the wide-scale implementation of AVs. The latter items included three relating to employment and skills. Respondents were asked to report how likely it is that the introduction of fully autonomous vehicles would result in “Job losses (e.g., truck and taxi drivers)”, “Loss in human driving skills over time”, and “Increased jobs (e.g., IT technicians)”.Given the recency of the advent of AVs and lack of available relevant survey instruments, more detailed information relating to perceptions of the implications of AVs for working life was obtained in Study B. This was achieved via a series of interviews with a broad range of stakeholders from sectors that will be impacted by the advent of these vehicles. Sectors represented by the interviewees included government (local, state, and federal departments responsible for health, transport, and/or infrastructure), trade unions, the law, technology firms, AV manufacturing/servicing companies, insurers (public and private), transport policy consortiums, and academia. In total, 38 interviews were conducted with 43 interviewees (3 interviews were conducted with multiple participants). The majority of the interviews (*n* = 28, 33 interviewees) were conducted in Australia, with the remaining 10 involving stakeholders in the US, the UK, France, and Sweden. The inclusion of international stakeholders permitted investigation of the extent to which the situation in Australia is reflected in international developments.

Recruitment was undertaken by a combination of (1) snowball sampling, whereby interviewees recommended other interviewees who would be relevant to the study [25] and (2) directly contacting individuals/organisations who were identified as potential key informants via news stories and internet searches. Ethics approval for the study was obtained from the Curtin University Human Research Ethics Committee (approval number HRE2017-0039) and all participants provided informed consent. Assurance was provided that interviewees’ contributions would remain anonymous.

Reflecting the diverse nature of the sample, the interviews were largely unstructured to enable each interviewee to talk at length about the way in which AVs are likely to emerge in their sector and the implications for their organisations and society in general. In 23 of the 38 interviews (15 Australian, 8 non-Australian), the topic of employment was raised as an issue that will need to be considered and carefully managed to optimise the positive outcomes of AVs while minimising any adverse consequences. These discussions formed the dataset analysed in the present study.

The interviews were audio-recorded and transcribed verbatim. The average length of the interviews was 67 min. The transcripts were imported into NVivo 11 for coding and analysis. A single researcher undertook the coding process due to the need to incrementally construct the coding framework to reflect the lack of *a priori* theory to guide code development and the diverse content of individual interviews. A combination of line-by-line coding and auto coding using common terms was used to allocate the transcript text to the coding framework. During the coding process, employment issues were identified as a substantial category within the data and a thematic analysis was conducted on the data relating to this topic. As such, a grounded approach was adopted that involved allowing primary issues of relevance to emerge from the data and analysing these issues to identify areas of commonality and divergence [26]. Triangulation in data collection and analysis was achieved by consulting a diverse pool of interviewees from multiple countries, discussing emerging interpretations with later interviewees to assess their utility, and reaching consensus within the research team [27].

## 3. Results

### 3.1. Quantitative Data from Study A (Public Opinion Survey)

Table 1 and Table 2 present the results from the items in the public opinion survey relating to the potential implications of AVs for jobs and skills. Respondents were asked to indicate their perceptions relating to work-related potential outcomes of AVs, specifically their concerns relating to job losses and the loss of driving skills, and the likelihood of more jobs becoming available. More than half (60%) of respondents reported being at least moderately concerned about job losses and almost three-quarters (71%) were at least moderately concerned about loss of driving skills. Around half (49%) believed that the introduction of AVs would result in increased jobs in technical areas.

### 3.2. Qualitative Data from Study B (Stakeholder Interviews)

Three primary themes were evident in interviewees’ discussions of the most likely potential employment impacts of the wide-scale implementation of AVs. These were consistent across countries and related to (1) the inevitability of the universal use of AVs and hence the need for immediate labour market planning around this issue, (2) the need to also recognise the extensive likely consequences for occupations that are not primarily structured around driving, and (3) potential positive effects in the form of worker safety and commuting opportunities. Each of these themes is summarised in Table 3 and discussed below with illustrative quotes provided.

#### 3.2.1. Theme 1: Planning for the Inevitable

The dominant response relating to the effects of AVs on employment was that the inexorable move towards automation in vehicles will bring substantial labour market changes resulting from the eradication of entire occupations that are based on driving (e.g., taxis drivers, road freight drivers, couriers, train drivers, and bus drivers). Parallels were often drawn with other technological innovations such as the sewing machine, electric streetlights, and the original motor car. The interviewees representing automotive and technology sectors in particular were consistent in their descriptions of AV technology as being well progressed and an inevitable feature of future transportation systems.

*I think it’s an inevitability, and I think it’s a good idea too. Are there going to be problems with the rollout of the new technology? Inevitably. Can they be sorted to everyone’s satisfaction? Eventually. So yes, it’s going to happen*.

Vehicle autonomy was described as both a major opportunity for productivity increases and a substantial challenge for the planning and management of individual workplaces and entire labour markets. The extent to which the positive outcomes can be optimised was seen as being dependent on the implementation of strategic initiatives designed to minimise negative disruptive outcomes. An important first step was noted to be ensuring that those whose jobs are likely to be made redundant understand the impending changes and their options for preparing for new roles.

*In terms of reskilling, if people don’t even know that they should start thinking about reskilling—it would be kind of good to make sure people are aware ahead of the game rather than losing their job and then figuring out that they’ve got to do something else*.

*It worries people. I think if when companies start to offer (automated transport) services, (it would be good) if they can give real concrete examples of how they’re (a) creating more jobs and (b) creating jobs that are still accessible to people at a whole range of different skill levels*.

The communication and re-skilling processes were generally seen to be manageable due to the likely decade or so of lead time before wide-scale implementation of AVs and the resulting opportunity to allow drivers to either retire out of the industry over time or train for other positions. Some noted that particular kinds of driving jobs tend to be transient (e.g., taxi or ride-share drivers who work in the sector either as an income supplement or while seeking work in other industries). Others commented that many driving jobs are unsatisfying and potentially unhealthy, making their eradication a positive outcome as long as other employment opportunities are available.

*Professional truck driving is one of the worst jobs on the planet, even in Australia, let alone in second and third world countries…it’s not going to happen for 10 years anyway…(and) it’s an aging workforce*.

*The freight industry—they have the biggest incidence of sleep apnoea and obesity. I actually think that because we need our truckies (truck drivers) to deliver our stuff and to get our stuff and all of us are really living wonderful lives because these guys at the very end of a supply chain are treated so badly. It’s the most dangerous job in the country and they’re not supported enough*.

A proposed alternative to drivers leaving the industry altogether was the conversion of driving jobs to other transport-related positions. At least in the short to medium term, it was envisaged that there would be a need for humans to remain involved in certain aspects of the transportation process. These aspects include receiving deliveries, communicating with delivery recipients, and providing assistance to passengers. There was a view that new roles developed around these tasks could be more diverse and enjoyable than many drivers’ existing jobs.

*There is also an opportunity for the service providers to still maintain those jobs but use them for better service provision. Like, for example, assisting people with disabilities rather than for the function of driving only*.

*The idea is maybe to train them in that new (AV) vehicle, to teach them how it’s running, and to train them in explaining to other people about the system. The idea is, later, maybe to be there, to control the vehicles, to see if everything is okay, to do maybe some kind of maintenance, to check if the service is running well, to be involved in the control monitoring side*.

It was also noted that passenger transport vehicles require ongoing servicing that will need to be undertaken by humans for the foreseeable future:

*Presumably these vehicles are still going to need to be husbanded. They are going to need to be cleaned and they’re going to need to be inspected. So jobs will be created by them. They just won’t necessarily be the drivers, it will be somebody else doing the maintenance*.

Finally, there was the suggestion that despite having the option to use AVs, there may be continuing demand for human-delivered transport services because of the innate need for human interaction and to engage in productive activity. For some interviewees, these needs will prevent, or at least considerably delay, the universal replacement of drivers.

*I think people need people. So that’s not going to go away in a hurry. People need jobs as well. So I think we’re still going to have a lot of that human element*.

A key element of suggestions for migrating employees out of a shrinking driver labour market was proactive planning to manage the incremental eradication of driving jobs to ensure that appropriate deployment strategies are enacted in a timely manner. The responsibility for developing and implementing these strategies was considered to lie across sectors, including governments, employers, and employees.

*It shouldn’t just be up to the workers to work out their own just transition. There really should be something on offer from governments, something on offer from employers who want to introduce these autonomous vehicles and all of those sort of things. (AVs are) sold just in terms of, “Look how much money we’ll save!”. But part of the package is that it should actually go not just to customers, not just to shareholders, but also their workers*.

#### 3.2.2. Theme 2: Effects on Other Occupations and Industries

There was a general understanding that the employment implications of the advent of AVs will be much broader than just the effects on driving occupations. Some interviewees envisaged positive outcomes in the form of the development of numerous new skilled roles, while others anticipated wide-scale job losses across associated sectors and the economy in general. These two contrasting viewpoints are outlined below.

In terms of job creation, it was expected that the introduction of AVs and their incremental advancement towards wide-scale use will result in many new jobs involving the development and implementation of the new technology. In most cases, these new positions were described as being highly skilled and rewarding.

*Of course it may remove some jobs. Okay, but how many other jobs it will create? How many new jobs will appear because of that technology? How many new jobs will be there to handle our AV? How many high-qualified jobs will rise from that technology in order to integrate it in the environment, to redefine cities, to use that technology in the most optimised way? It will bring a lot of new jobs*.

It was acknowledged that these new positions are likely to require combinations of skills that are not common in the current workplace, necessitating an evolution in training to accommodate the labour needs of future organisations.

*It really brings about a new breed of people that you need. Because it’s not an IT thing and it’s not a functional expertise thing. It’s a hybrid person that understands processes and has some IT knowledge. So it’s people that can do modelling. It’s a core skill set that isn’t prevalent broadly in a lot of organisations. So, this whole automation of business process is going to breed, I think, a whole new cadre of people that there’s not a lot of today, who really understand enough about a process and how it all works, and then understand technology*.

In terms of job losses in other sectors, numerous employment categories were nominated as being especially vulnerable to replacement by AVs in the shorter term. Along with driving occupations, interviewees mentioned occupations as diverse as farmers, personal assistants, and volunteers who assist the elderly and disabled. The following interviewee explained how AVs are already performing the roles previously undertaken by farmers:

*We have a machine that travels the cropped fields. It’s just four wheels, it’s got a tank of herbicide on it, and it’s got a manual arm. It goes along and picks the weed out from between the crops, so it knows the difference between the crop and the weed. First of all it does it in the manual and safer sense, but if it can’t get it, it will squirt a little herbicide on it and kill it. Also, if you want it to, it will also squirt some fertiliser into the plant. Now that’s run with a solar cell. It’s worth $30,000… So that $30,000 piece of equipment, really does the farmer’s job. And then they have the same for fruit trees and things like that…So that sort of autonomy piece is really there in agriculture*.

The implications for volunteering positions were discussed in the context of many such positions involving assisting the elderly and disabled with their transport needs, such as taking them to and from medical appointments and assisting with grocery shopping. Where the primary role of the volunteer is to drive a vehicle, it was expected that AVs would reduce the demand for volunteer services. Where the assisted individual is in need of other services (e.g., getting in and out of the vehicle), it was noted that it may be some time before AVs have evolved to the point where they can also provide this level of assistance.

*There would be a whole lot of volunteers out of jobs down at the hospital because there’s a whole sort of volunteer movement around moving people to health appointments. That potentially goes, if they can just get into an autonomous vehicle. Sometimes they’ll still need a second person to come with them…If you have a physical disability you may still need a second person to help you. But if it’s a sight disability, it opens up a whole new world for you*.

At a broader level, some interviewees expressed concerns about the effects of AV on entire economies as numerous categories of jobs disappear through increasing automation.

*It’s all very George Jetson and it looks very appealing if we can get there without making mankind completely redundant in the meantime and have no jobs and no money to spend on these products that have got to be transported…What’s the meaningful role for people in a society that’s highly automated, which is where we’re now - we thought we were automated. We’re now about to flick over. That will increasingly become the case for places like China and India as well. The manufacturing hubs now are going to have to find new things for their staff to do, for people to do, to give them meaningful roles in life*.

#### 3.2.3. Theme 3: Other Employment-Related Outcomes

The central role of transport in modern life was discussed in terms of people having adequate work/commute alternatives. This was noted as being especially important for those who may lack the income or proximity to city centres to enable them to engage in the travel necessary to hold down a job. AVs were described as having the potential to provide convenient and cost-effective transport for work commutes.

*The issue that impacts people on transport is in fact their income. Lower socioeconomic groups - transport is really big for them, getting access to transport. If you’ve got a job that requires you to travel an hour or two in traffic, you’d want to get the cheapest way there*.

*If you can get people mobile so that they can get to employment or get to where they need to be, that is massive*.

It was noted that members of highly disadvantaged groups may have the most to gain from AVs through the benefits associated with access to employment and the accompanying sense of purpose in life.

*It’s a huge problem for Indigenous populations around access to vehicles. In fact they’re often in jail because they drove without a licence and getting a licence is often very difficult for them. There’s the whole issue of barriers to employment, opportunities, what they then do…those things are really significant for Indigenous communities*.

The ability to use AVs for the work commute was also discussed in terms of permitting individuals to use their travelling time more pleasantly or productively, especially for those facing long trip times.

*I can definitely see the appeal for someone who has a really long commute and there are lots of places in the world where people have one-, two-hour commutes*.

There may also be the potential for AVs to increase worker safety. This could occur through the enhanced ability of AVs to avoid accidents (such as when workers need to interact with moving vehicles in the workplace) and to perform tasks that constitute unnecessary risks for humans:

*They’re also using drone trials (on farms). For example, if it’s a half hour drive to the other side of his property, he’ll send the drone out and he can see if the gate’s open, and probably use electronics—some sort of electronic mechanism, to close the gate*.

However, it was noted that a lack of other human workers on sites may leave individuals at increased risk.

*On mine sites, even if you’ve replaced all the drivers, there are still other human beings there. To what extent are they placed in danger by leaving a lot of their safety up to an AI effectively?*

## 4. Discussion

The survey results indicate that the general public has some appreciation of the extensive job losses that are forecast to eventuate in specific industries involving driving as a primary skill set [4]. Consistent with the findings of the stakeholder interviews, there also appears to be some recognition among the general public that new employment opportunities will emerge in a range of supporting areas. The view that AVs will both destroy and create jobs is reflected in conceptual discussions in the literature on the issue of employment outcomes associated with advances in technology [28,29,30,31].

Emphasis was placed in the stakeholder interviews on the need to proactively manage the transition to AVs to minimise the disruptive effects of inevitable job losses in driving and related occupations. This is aligned with recommendations to involve employees in discussions about the introduction of technology into the workplace to minimise the anxiety associated with job insecurity [32]. Such discussions could include consideration of the potential evolution of roles involving customer/passenger liaison that can utilise some of drivers’ existing skills in the interim phase prior to full-scale automation. Research suggests that at least initially, passengers will be more willing to use AV transport options if an attendant is present [33]. In the meantime, the study results highlight the importance of training future workers for new hybrid positions that will require unique combinations of skills. These positions are unlikely to be suited to re-trained vehicle operators [4], and hence will necessitate the development of new training and recruitment programs designed to equip future workers with the needed expertise.

Some of the identified effects of the wide-scale introduction of AVs may be beneficial for individuals and society in general. For example, the shrinking number of available volunteers [34] means that a reduced requirement for volunteer drivers may assist the distribution of the limited number of volunteers among positions where they are most needed. In addition, the availability of automated transportation can benefit low-income workers, for whom the availability of low-cost options for the work commute is especially important [35].

The largely exploratory and emergent nature of the methodological approach employed in the present study reflects the small body of prior work focusing on stakeholders’ awareness and understanding of the effects of autonomous vehicles on workers’ lives. This methodological approach precluded the *a priori* application of specific theoretical frameworks to the study design; however the findings can be assessed in terms of their relevance to existing concepts. The extended Technology Acceptance Model (TAM [36]) appears to have particular utility for interpreting the study results in terms of the implications for AV implementation processes. The extended TAM highlights the extent to which beliefs about the benefits of new technologies influence adoption decisions, including in the workplace. The acceptance of the inevitability of large-scale job losses in driving-related occupations among both the survey respondents and the interviewees reflects the clear advantages of AVs for these kinds of tasks. The TAM also includes variables relating to subjective norms, ease of use, demonstrability, and the extent to which individuals have experienced the technology as predictors of uptake. As AVs become more commonly used, their acceptability can be expected to increase due to greater observation opportunities and direct personal experience. This is likely to be relevant to the specific usage scenarios identified in the present study (i.e., AV use in occupations directly and indirectly related to driving and AV use for accessing the workplace), as well as across society in general. This higher level of acceptability may make the implementation of AVs in and around the workplace more commonplace and routine over time.

This study has limitations that can be addressed in future research. The survey was national in scope and hence could be replicated in other geographical contexts to assess the extent to which the identified anticipated effects on employment are common elsewhere. To some extent this was addressed by the second study phase involving an international sample of interviewees, the findings of which supported the quantitative results. However, the qualitative sample largely comprised Australian interviewees and future work could collect data from a wider range of countries. Sourcing survey respondents via a web panel provider constitutes a further potential limitation due to the lack of random recruitment. More generalisable data could be generated through the use of alternative participant recruitment methods.

## 5. Conclusions

The results of this study suggest that it is timely for governments and employers to communicate with workers about impending AV technology, the ways in which the associated changes are likely to impact industries and individuals, and the plans in place to manage this process. This in turn will require strategic efforts to conceptualise and plan for labour markets characterised by high levels of automation within and across industries. This is a highly challenging task that will require intensive collaboration between societal actors including government, industry, the not-for-profit sector, and the general community.

## Figures and Tables

**Table 1:** Public opinion survey results—Job/skill losses.

Issue	Mean ^1^	Extremely Concerned (%)	Very Concerned (%)	Moderately Concerned (%)	Slightly Concerned (%)	Not at all Concerned (%)	Don’t Know (%)
Concern about job losses (e.g., truck and taxi drivers)	2.96	17	18	25	20	14	7
Concern about loss of human driving skills	2.60	22	26	23	16	8	5

^1^ Mean on 5-point scale with ‘Don’t know’ responses excluded.

**Table 2:** Public opinion survey results—Job gains.

Issue	Mean ^1^	Very Unlikely (%)	Somewhat Unlikely (%)	Neither Likely nor Unlikely (%)	Somewhat Likely (%)	Very Likely (%)	Don’t Know (%)
Likelihood of increased jobs (e.g., IT technicians)	3.46	7	11	25	30	19	8

^1^ Mean on 5-point scale with ‘Don’t know’ responses excluded.

**Table 3:** Stakeholder interview thematic analysis results.

Theme 1: Planning for the Inevitable	Theme 2: Other Occupations and Industries	Theme 3: Other Employment-Related Outcomes
Driving occupations will disappear	Job creation	Access to employment
Job redesign opportunities	Job losses	Work commute
Strategic approach needed	Volunteering implications	Worker safety
Responsible parties	Suppression of economic activity	
